# Surface and Structural Characterization of PVTMS Films Treated by Elemental Fluorine in Liquid Perfluorodecalin

**DOI:** 10.3390/ma16030913

**Published:** 2023-01-18

**Authors:** Nikolay A. Belov, Aleksandr Y. Alentiev, Dmitrii S. Pashkevich, Fedor A. Voroshilov, Edgar S. Dvilis, Igor P. Asanov, Roman Y. Nikiforov, Sergey V. Chirkov, Daria A. Syrtsova, Julia V. Kostina, Yulia G. Bogdanova

**Affiliations:** 1 Tomsk Polytechnic University, 30, Lenin Avenue, 634050 Tomsk, Russia; 2 A.V. Topchiev Institute of Petrochemical Synthesis, Russian Academy of Sciences, 29, Leninskii Prospect, 119991 Moscow, Russia; 3 Institute of Applied Mathematics and Mechanics, Peter the Great St. Petersburg Polytechnic University, 29, Polytechnicheskaya St., 195251 Petersburg, Russia; 4 A.V. Nikolaev Institute of Inorganic Chemistry, Siberian Branch of Russian Academy of Sciences, 3, Academician Lavrentiev St., 630090 Novosibirsk, Russia; 5 Chemical Department, M.V. Lomonosov Moscow State University, GSP-1, Leninskie Gory, 119991 Moscow, Russia

**Keywords:** direct surface liquid-phase fluorination, PVTMS, SEM, XEDS, XPS, ATR-IR, wetting method

## Abstract

Poly(vinyl trimethylsilane) (PVTMS) films were subjected to direct surface fluorination in liquid medium (perfluorodecalin). The samples were investigated using several techniques: SEM-XEDS, XPS, ATR-IR, and contact angle measurement. The methods used allowed us to estimate chemical changes occurring because of the treatment. ATR-IR showed that most of the changes occurred in the Si(CH_3_)_3_ group. Monofluorinated Si(CH_3_)_3_ groups formed in the near-surface layer (Ge crystal, 0.66 µm penetration) after 30 min of fluorination, and then di- and trifluorinated groups appeared. Oxidation of the film with oxygen was also shown with the use of ZnSe crystal (2 µm penetration). The XPS method allowed an assessment of the ratio of the main elements at the surface of the fluorinated film. Two different exponential models were proposed to fit the experimental data of SEM-XEDS. Based on the model with the intercept, the depth of fluorination was estimated to be ≤1.1 µm, which is consistent with the result from the literature for the gas-phase fluorination. Contact angle measurements showed that oxidation of the PVTMS surface prevailed for the first 45 min of fluorination (surface hydrophilization) with a subsequent fluorine content increase and hydrophobization of the surface upon 60 min of fluorination.

## 1. Introduction

PVTMS is a well-known polymer for membrane gas separation with relatively high gas and vapor permeability (*P*(O_2_) = 40 Barrer (1 Barrer = 10^−10^ cm^3^(STP)·cm·cm^−2^·s^−1^·(cmHg)^−1^)) and gas selectivity [1]. It had commercial applications several decades ago and was considered a polymer with satisfactory performance, but nowadays, PVTMS can hardly compete with the modern high-performance polymers [2,3,4]. Nevertheless, PVTMS membranes are intensively studied in pervaporation and vapor-phase separation [5] or liquid separation processes [6], i.e., the processes that depend highly on the surface properties of a membrane. The modified surface can be heavily involved in interactions with penetrating molecules and, thus, can affect the separation properties of the polymeric membranes. For instance, hydrophilic membranes are utilized for the removal of water from solvent–water mixtures (solvent dehydration) while hydrophobic membranes are used for the selective permeation of the solvent from solvent–water (solvent recovery) and solvent-organic (organic–organic separation) mixtures [7]. The hydrophobic–hydrophilic balance of the surface is usually estimated by a wetting method, which is an informative method to assess surface property changes as the contact angle is sensitive to the chemical composition of the surface [8,9].

To change surface properties and improve the separation characteristics of PVTMS-based membranes, various modification techniques were previously applied: radiation grafting [10], heat treatment [11], plasma treatment [12,13], and gas-phase fluorination [14,15,16]. It was shown that some types of treatment, i.e., heat treatment and fluorination, are promising for stability, permeability, and selectivity improvement. Direct fluorination was demonstrated to significantly change the chemical composition of the polymeric surface, forming super-hydrophobic or hydrophilic materials [17].

As the new method of polymer modification, direct surface liquid-phase fluorination was recently developed to provide fluorination under mild conditions in comparison with those for gas-phase fluorination [17]. While it is expected that the replacement of H atoms with F atoms will lead to Teflon-like surface formation, when mild fluorination is performed, polar groups may change their orientation towards the surface and surface hydrophilization occurs [18,19]. In addition, the fluorination may be associated with oxidation due to the interaction of oxygen with long-lived active radical centers appearing during the fluorination process, resulting in an increase in oxygen-containing groups and subsequent surface hydrophilization [17]. Therefore, it is a huge challenge to estimate the chemical composition of the fluorinated layer, to assess the depth of fluorination, and to characterize a modified material in terms of hydrophobicity/hydrophilicity.

Thus, the present work is dedicated to the investigation of PVTMS film treatment with elemental fluorine in liquid medium (perfluorodecalin) and the characterization of the treated film using a series of physico-chemical methods: attenuated total reflectance infra-red spectroscopy (ATR-IR), scanning and energy-dispersive X-ray spectroscopy (SEM-XEDS), X-ray photoelectron spectroscopy (XPS), and contact angle measurement.

## 2. Materials and Methods

### 2.1. Preparation of PVTMS Samples for Fluorination

The PVTMS film was prepared by redeposition from the commercial PVTMS membrane (manufactured in the USSR at the Kuskovo chemical plant). The PVTMS membrane was dissolved in toluene to obtain a 3 w/w% solution. The solution was filtered into a molding ring with a cellophane bottom using a pipette with a cotton wool in the tip. After evaporation of the solvent, the film was carefully removed from the ring and dried under a vacuum (~1 mbar) to a constant weight. All samples were placed into plastic bags and sealed to avoid water uptake and to prevent rapid aging. The thickness of the films was measured by a standard micrometer (±0.25 µm) and corresponded to ~40 μm.

### 2.2. Direct Liquid-Phase Fluorination of PVTMS Samples

Surface fluorination of PVTMS was carried out according to the following procedure. A gaseous mixture of fluorine (10 vol.%) and nitrogen (90 vol.%) was bubbled into a chamber made of fluoroplast (hereinafter the reactor) with perfluorodecalin (PFD) at room temperature (22 ± 2 °C) for 60 min to saturate the liquid phase with fluorine and simultaneously remove dissolved air oxygen. The flow rate was set to 0.5 cm^3^·s^−1^ using a flow meter Bronkhorst F-201EV-AAD-33-K. A magnetic stirrer allowed the mixing of the liquid phase. After the specified time, the fluorination mixture supply was stopped. The membranes were then treated by fluorine dissolved in PFD at room temperature (22 ± 2 °C). A sample of PVTMS was placed into a holder (the diameter of the films was 47 mm) and installed into the reactor with PFD in such a way that surface liquid-phase fluorination took place on one side. The fluorination mixture was supplied (with a flow rate of 0.5 cm^3^·s^−1^) to the reactor while mixing with the magnetic stirrer. The experiment was held at a room temperature. After 15 min of processing, the fluorination mixture supply was stopped and the magnetic stirrer was turned off. The sample of PVTMS (F-PVTMS-15) was taken out of the reactor and dried with filter paper. The surface fluorination of PVTMS with the fluorination mixture was also carried out for 30 (F-PVTMS-30), 45 (F-PVTMS-45), and 60 (F-PVTMS-60) min. The experiment was carried out for two films for each fluorination time.

A control sample of PVTMS for SEM/XEDS analysis was prepared in the reactor without a supply of the fluorination mixture. The PFD was stirred for 30 min using the magnetic stirrer, and the surface layer of the PVTMS film was saturated with PFD. After 30 min, the membrane holder was removed from the reactor and the PVTMS film control sample (F-PVTMS-0-PFD) was taken out. The excess PFD on the surface of the PVTMS film was removed by placing the film between two discs of filter paper.

### 2.3. X-ray Photoelectron Spectroscopy (XPS)

The surface chemical composition of the samples was studied using a FLEXPS X-ray photoelectron spectrometer (Specs, Germany). The spectrometer is equipped with a Phoibos 150 hemispherical electron analyzer and an electron detector with a 150-channel plate and a 1-DLD delay line. The film samples were attached to a molybdenum holder using double-sided tape. The spectra were recorded from the side of the fluorinated layer. Excitation of the spectra was carried out by Mg Kα radiation from an X-ray tube with a dual anode XR-50. The transmission energy of the electron analyzer when recording the panoramic spectra was 50 eV, while for individual lines it was 20 eV. The measurements were carried out at room temperature; the vacuum in the system when recording spectra of the samples was ~10^−9^ mbar. The changes in the line shapes caused by the degradation of polymer samples under X-ray irradiation were observed during the spectra recording, so the shortest measurement time was set to obtain reliable statistics. The results of the average spectra are provided.

The electron binding energy was measured relative to the reference: relative to the Si2p line = 100.5 eV and C1s = 284.4 eV in the virgin sample [20] and, accordingly, for fluorinated samples relative to the line C1s = 284.4 eV of carbon atoms of the component with the lowest binding energy.

The relative atomic concentrations of elements were calculated from the peak area, taking into account the electron photoionization cross-section, the electron free path, and the electron transmission function. The size of the analyzed section of the sample was about 2 mm. The spectra were decomposed into symmetric components simulating convolution of Lorentz and Gaussian lines for detailed analysis. The background of inelastic electron scattering was subtracted using the Shirley method. The satellite lines Mg Kα_3,4_ were subtracted from the X-ray excitation spectrum before decomposing the spectra into components. The decomposition of the spectra was carried out using the CasaXPS program. An IQE 12/38 ion etching gun was used to perform the layer-by-layer analysis of the PVTMS films. An Ar^+^ ion beam with an energy of 1 keV was used with a surface scan of 5 × 5 mm^2^. The ion current was 29 µA. An ion etching cycle was carried out for 60, 180, 300, 600, and 900 s for each sample; the XPS spectra were recorded after each etching.

### 2.4. Scanning and Energy-Dispersive X-ray Spectroscopy (SEM and XEDS)

Cross-sections of the PVTMS film samples were prepared prior to the analysis. The samples were stacked into a single pack with the same orientation of the fluorinated sides. The cross-section surfaces were coated with platinum to ensure an electron sink during SEM analysis. The SEM analysis was performed using a JSM-7500FA (JEOL, Akishima, Japan) microscope. The XEDS analysis was performed using an XFlash 6|60 (BRUKER, Berlin, Germany) device.

### 2.5. Attenuated Total Reflectance Infra-Red Spectroscopy (ATR-IR)

ATR-IR spectra of the fluorinated PVTMS films were recorded in the mode of attenuated total reflectance (ATR) on an IFS 66 v/s IR Fourier spectrometer with an ATR instrument (with Ge or ZnSe crystal) in the region of 4000–600 cm^−1^, with a resolution of 2 cm^−1^ and averaged over 50 scans. Ge crystal has a lower radiation penetration depth, which allows analysis of the changes in the chemical structure at a depth of up to 0.66 µm. Thus, the Ge crystal was used to perform a more thorough study of the processes occurring in the near-surface layer of the PVTMS film during its fluorination.

### 2.6. Contact Angle Measurements and Surface Energy Calculation

The advancing contact angles (*θ*) of two test liquids (H_2_O, CH_2_I_2_) were measured at 20 ± 1 °C using a horizontal microscope equipped with a goniometric attachment. The dispersion (*γ^d^_SV_*) and polar (*γ^p^_SV_*) components of the surface free energy (*γ_SV_*) values of the composite films were determined by the two-fluid method within the framework of the Owens–Wendt–Kaelble approximation [21,22]. No less than 6 drops of probe liquids were used for calculating the average values of the contact angles. The measurement accuracies were 1° for contact angles and 1 mJ∙m^–2^ for the surface and interfacial energies of the polymer films.

## 3. Results and Discussion

### 3.1. ATR-IR Analysis of the PVTMS Samples

**Figure 1:**
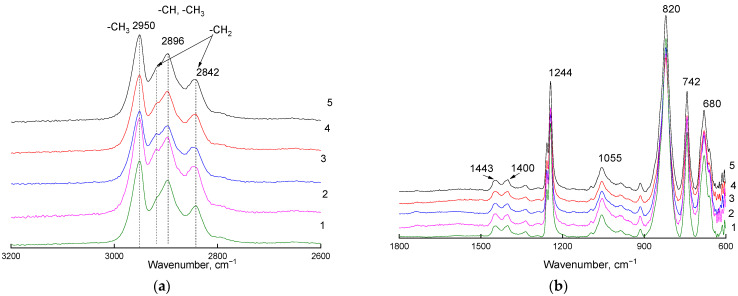
Fragments ((**a**): 2600–3200 cm^−1^ region; (**b**): 600–1800 cm^−1^ region) of ATR-IR spectra (ZnSe crystal) of the PVTMS film recorded on the side opposite to the flow of the fluorinating agent. Curve 1: original film; curves 2–5: films fluorinated for 15, 30, 45, and 60 min, respectively.

Figure 1a shows a fragment of the ATR-IR spectra (ZnSe crystal) of the PVTMS film recorded on the side opposite to the flow of the fluorinating agent, where –CH_3_, -CH_2_, and -CH stretching vibration absorption bands are located. Figure 1b shows a fragment of the same spectra, where bending vibration absorption bands are located as well as absorption bands assigned to the Si-C bond. There were no changes in the position, number, and relative intensity of the absorption bands of the main functional groups of PVTMS.

**Figure 2:**
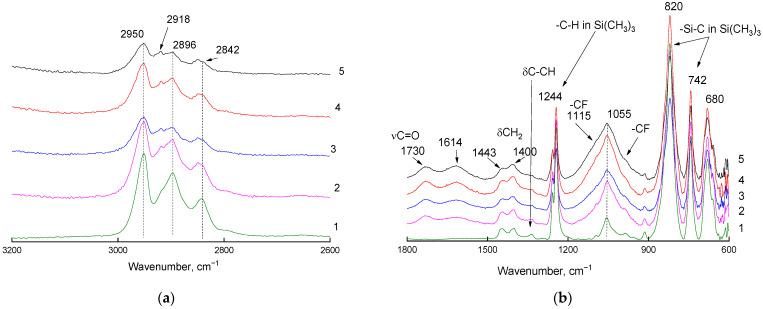
Fragments ((**a**): 2600–3200 cm^−1^ region; (**b**): 600–1800 cm^−1^ region) of the ATR-IR spectra (ZnSe crystal) of the PVTMS film recorded on the side of fluorination. Curve 1: original film; curves 2–5: films fluorinated for 15, 30, 45, and 60 min, respectively.

Changes in the IR spectra of the films were observed on the side of the fluorination (Figure 2). The pattern of the ATR-IR spectrum changed in the absorption region of the C-H bonds stretching vibrations for 15 min of fluorination (Figure 2a). A decrease in the intensity of the absorption band of the CH3 group stretching vibrations (2950 and 2896 cm^−1^) in the Si(CH_3_)_3_ fragment with a simultaneous increase in the relative intensity of A_2920_/A_2896_ indicates a decrease in the number of methyl groups in the trimethylsilyl group relative to the methylene groups. In case of gas-phase fluorination, it was previously shown in the work conducted by Kharitonov et al. [15] that CH, CH_2_, and CH_3_ bands were absent from the spectra when fluorination was performed using undiluted fluorine. Therefore, the formation of C-F_x_ bonds (1000–1400 cm^−1^) and the probable formation of Si-F (848 cm^−1^) and C-SiF_3_ (1010 cm^−1^) bonds were observed. Thus, this confirms milder fluorination of PVTMS in the case of liquid-phase fluorination compared to gas-phase fluorination.

The changes were symbatic in the middle region of the ATR-IR spectra; 15 min after the start of fluorination, the ratio of the intensities of the absorption bands of the bending vibrations of δCH_2_ (1443–1440 cm^−1^) changed: the half-width and intensity of the absorption band increased at 1055 cm^−1^ due to short- and long-wave shoulders characterizing the C-F bonds. Moreover, these changes were accompanied by a decrease in the intensity of the absorption band at 1340 cm^−1^ (bending vibrations of the C–CH bond in the backbone) and the appearance of additional absorption bands at 1730 and 1614 cm^−1^. A slight increase in the intensity in the high frequency region was also observed (above 3400 cm^−1^, not shown in Figure 2). Taking into account the presence of small amounts of oxygen and water in the fluorinating agent, it is impossible to exclude (i) the oxidation of PVTMS with the cleavage of the tertiary hydrogen atom from the backbone with the formation of a carbonyl group and (ii) the sorption of water in the surface layer of the film, including due to hydrogen bonds HOH---O=C(CH_2_)–. Carbonyl group formation was also observed in the spectra of gas-phase fluorinated PVTMS in the work conducted by Kharitonov et al. [15].

**Figure 3:**
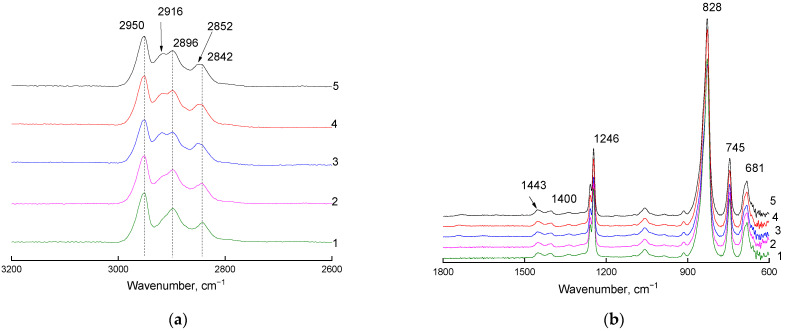
Fragments ((**a**): 2600–3200 cm^−1^ region; (**b**): 600–1800 cm^−1^ region) of ATR-IR spectra (Ge crystal) of the PVTMS film recorded on the side opposite to the flow of the fluorinating agent. Curve 1: original film; curves 2–5: films fluorinated for 15, 30, 45, and 60 min, respectively.

**Figure 4:**
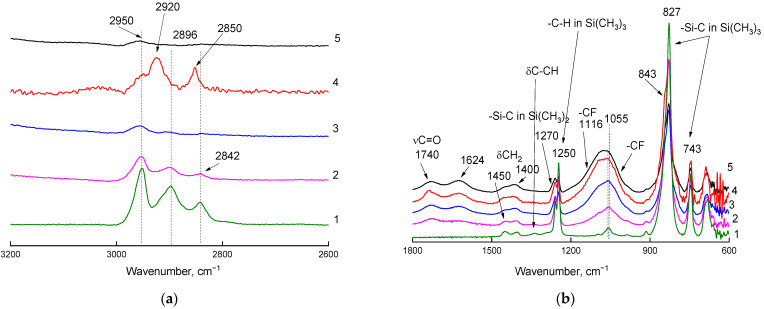
Fragments ((**a**): 2600–3200 cm^−1^ region; (**b**): 600–1800 cm^−1^ region) of ATR-IR spectra (Ge crystal) of the PVTMS film recorded on the side of fluorination. Curve 1: original film; curves 2–5: films fluorinated for 15, 30, 45, and 60 min, respectively.

ATR-IR spectra of the same films were also recorded on the Ge crystal (Figure 3 and Figure 4). The formation of C–F bonds was not observed (Figure 3b) on the side opposite to the flow of the fluorinating agent.

The changes were more significant on the side of fluorination and, in general, coincided with the changes described above for the ATR-IR spectra recorded on the ZnSe crystal.

The appearance of the shoulder at 1270 cm^−1^ on the absorption band of the H–C–H bending vibrations in methyl groups (1240–1250 cm^−1^) can be attributed to the appearance of Si(CH_3_)_2_ groups, which may indicate either fluorination of the Si(CH_3_)_3_ at one of the methyl groups or the methyl group cleavage from the Si(CH_3_)_3_.

Two intense absorption bands in the long–wavelength region of the spectrum (at 820 and 740 cm^–1^) belong to the Si–C stretching vibrations, with the Si–CH_3_ bond making a larger contribution to the first band, and the Si–CH bond of the polymer backbone making a larger contribution to the second band. The appearance of short–wave shoulders on these absorption bands allows one to conclude that only methyl groups within Si(CH_3_)_3_ were fluorinated during the first 30 min of fluorination, and only monofluorinated groups formed; then difluorinated (changes in the region of 1240 cm^−1^) and trifluorinated (bands in the region of 1400 cm^−1^) groups began to form. Silicon fluorination cannot be excluded when the deep fluorination process is performed.

The complexity of the semi-quantitative assessment consists of choosing an absorption band that can reasonably be used as an internal standard. Fluorination changes the polarity of all neighboring functional groups, and the fairly simple structure of the polymer chain excludes the presence of “stable” fragments whose vibrations could have been reflected in the spectrum without changing the spectral characteristics of the absorption bands.

### 3.2. XPS Analysis of the PVTMS Samples

Unlike the other methods used in the present paper, XPS analysis allowed us to investigate the very surface of the film (<1 nm). Thus, it is useful to study chemical changes that occur on the surface of the fluorinated film.

**Figure 5:**
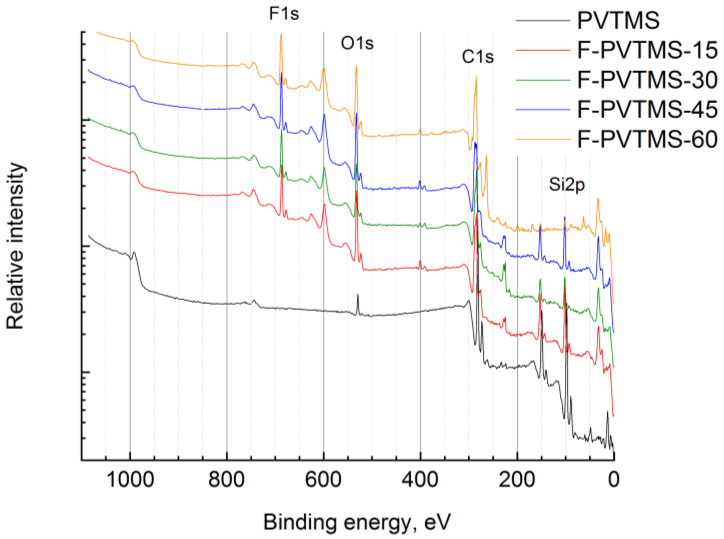
The overall XPS spectra of the surface of the virgin and the fluorinated PVTMS samples.

C, Si, and O elements were detected in the virgin sample (Figure 5). The ratio of C/Si was 4.0, and that of O/Si was 0.15. C, O, F, Si, as well as impurities of N (~1–2 at. %) and Zn (~0.1 at. %), were found in the fluorinated samples.

**Figure 6:**
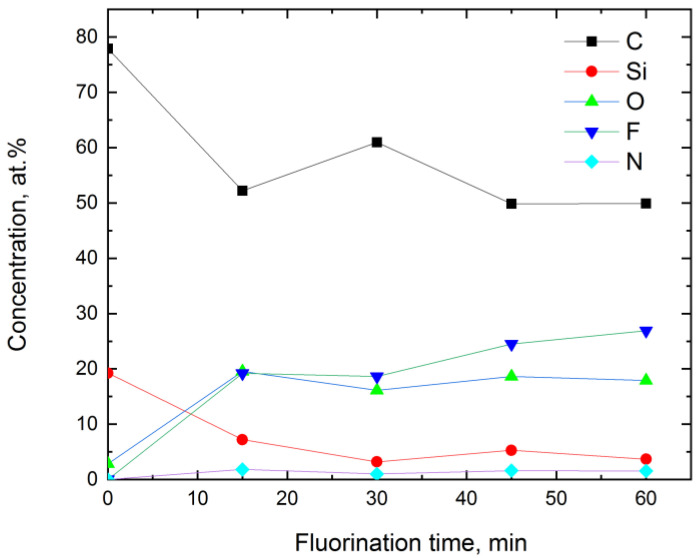
Change in the concentration of elements on the surface of PVTMS samples determined by XPS depending on the fluorination time.

Figure 6 shows that an increase in the fluorination time led to the increase in the concentrations of F and O, while the concentrations of C and Si decreased, and the nitrogen concentration stayed practically the same (1.5–1.8 at. %). However, there were some peculiarities observed in Figure 6. The carbon content on the very surface of the fluorinated samples decreased sharply from 78 (virgin PVTMS) to ~50 at. % for the samples fluorinated for 15 min and longer. This decline may be due to possible cleavage of C-Si moieties during fluorination. Meanwhile, a similar decrease was also observed for silicon atoms: from 20 (virgin PVTMS) to 3–5 at. % for the fluorinated samples.

The surface of the PVTMS samples was fluorinated significantly (up to 20 at. %) starting with the sample treated for 15 min (Figure 6). Further fluorination resulted in a slight gradual increase in the fluorine content up to ~25 at. %. The C/F ratio was close to ~2 for the samples fluorinated for 45 and 60 min; however, this parameter may be not reliable because of the possible cleavage of the C-Si bond and the corresponding removal of carbon atoms from the PVTMS chemical structure. XPS data also proved that the fluorination procedure was accompanied by oxidation. The oxygen content in the polymer surface reached 16–20 at. %, and the C/O ratio reached 2.7–3.8, i.e., the amounts of oxygen and fluorine atoms in the repeat unit of the polymer were comparable. Significant oxidation was previously observed for liquid-phase treatment of poly(2,6-dimethylphenyleneoxide-1,4) [23] and metathesis-based polynorbornene [24] with the elemental fluorine.

According to the C/Si ratios observed for the PVTMS samples treated for 45 and 60 min, one can assume that three to four Si atoms (i.e., Si(CH_3_)_3_ groups) in the five repeat units escaped the structure. Therefore, five repeat units on the very surface of the PVTMS films fluorinated for 45 and 60 min contained 13 C, 1 Si, ~6 F, and ~5 O atoms.

**Figure 7:**
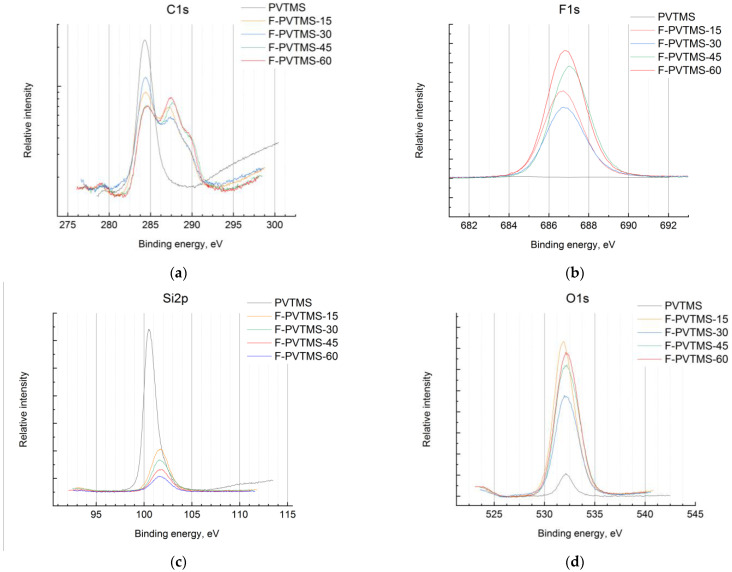
XPS spectra of C1s (**a**), F (**b**), Si2p (**c**), and O1s (**d**) lines for the control sample (PVTMS) and PVTMS films fluorinated for 15 (F-PVTMS-15), 30 (F-PVTMS-30), 45 (F-PVTMS-45), and 60 min (F-PVTMS-60).

Analysis of the binding energies of the elements on the XPS spectra allowed us to make a detailed estimation of the chemical structure of the fluorinated layers. The C1s spectrum (Figure 7a) of virgin PVTMS was a single line with an energy of C1s = 284.3 eV. The Si2p (Figure 7c) decomposed into two lines with energies of 100.5 eV of the Si-C bonds and a wide line of lower intensity at 101.4 eV of the Si-O bonds. The O1s (Figure 7d) line was a single line with an energy of 532.1 eV of O-Si bonds. Therefore, 4–5 at. % of oxygen in the virgin PVTMS film existed in the bounded Si-O form. The lines of C1s, Si2p, and O1s widened when the ion etched due to the destruction of chemical bonds.

The C1s spectrum of fluorinated samples contained four components at 284.4, 285.8, 287.5, and 289.5 eV, associated with C-C/Si, C-O, C-F, and O-C=O fragments, respectively. The component associated with the C-O bonds was ~15% and appeared at fluorination times of more than 15 min. The C-F component increased with the fluorination time. The component at 289.5 eV also increased. These findings support the ATR-IR results described in the section above. The F1s spectrum (Figure 7b) was represented by a single line at 686.7 eV (C-F bonds). The Si2p spectrum of the fluorinated samples was a single line at 101.7 eV, which was more than 1 eV higher than the value for the virgin PVTMS. An additional component appeared at 102.3 eV for fluorination times of more than 30 min. The O1s spectrum consisted of a main component at 531.7 eV and a weak line at 533.2 eV, the intensity of which increased with the fluorination time.

Treatment of the film surface by a Ar^+^ ion beam allowed us to monitor the chemical composition of the deeper layers of the films. The estimation of the depth of etching and scattering of the chemical composition is often unclear and depends on many effects (inorganic or organic nature of the surface, time of sputtering, energy of the beam, etc.) [25,26]. Therefore, an interpretation of the data obtained requires some assumptions. Hofstetter and Vaynzof [26] showed that, in spite of the different chemical natures of the sulfur-containing polymers that are frequently used in organic electronic devices, an average etching rate was about 1 nm per 15–20 s when using monoatomic Ar^+^ ion etching sources. Therefore, the mean depth of etching in the case of PVTMS-fluorinated samples treated for 60, 180, 300, 600, and 900 s appeared to correspond to 4–6, 9–12, 15–20, and 45–60 nm, respectively.

The sputtering duration dependences of the concentrations of carbon, silicon, fluorine, and oxygen atoms calculated based on the XPS spectra of the C1s, Si2p, F1s, and O1s lines are presented in Figure S1. The contribution of lines in the C1s spectrum decreased at 287.7 (C-F) and 289.3 eV (O-C=O). The contribution of the component at 284.4 eV (C-C/Si) increased. In the O1s spectrum, the component at ~531 eV disappeared and the line associated with Si-O remained at ~533 eV after ion etching. The Si2p spectrum after ion etching mainly contained the line at 103.5 eV of Si-O, but the contribution of Si-C bonds remained at 101.7 eV. The fluorine content gradually decreased to 10–15 at. % as the etching time increased to 900 s, that is, half of the initial fluorine concentration (Figure S1c). This fact clearly shows that the depth of fluorination was much greater than 45–60 nm even for the sample fluorinated for 15 min. The changes in the concentrations for the other elements with increased sputtering time proved the assumption. Therefore, the oxygen, silicon, and carbon contents (Figure S1a,b,d) did not reach their bulk concentration corresponding to that for the virgin PVTMS sample.

### 3.3. SEM and XEDS for the PVTMS Samples

**Figure 8:**
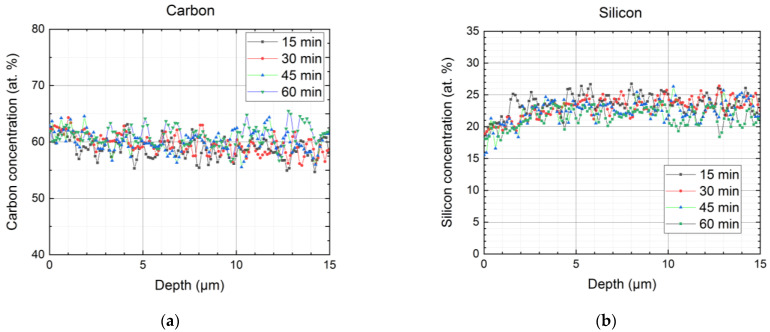

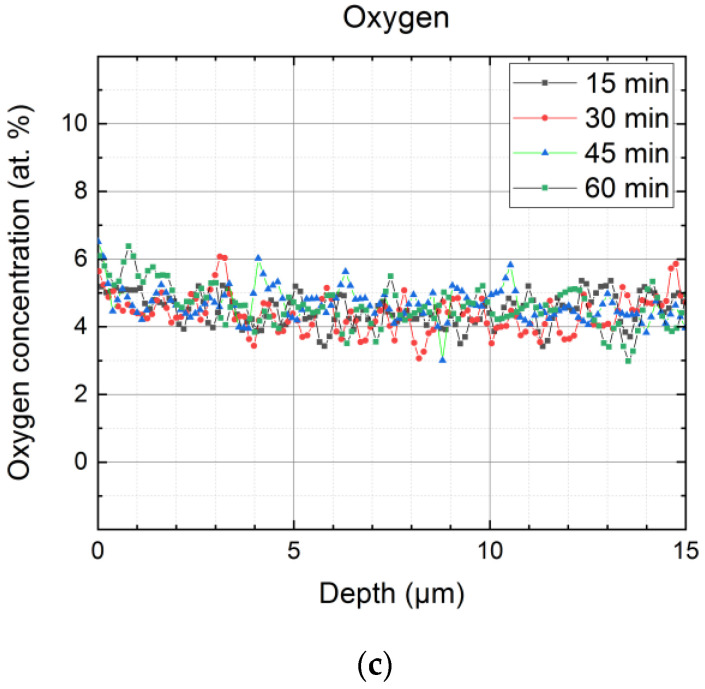
Carbon (**a**), silicon (**b**), and oxygen (**c**) content distribution over the thickness of the PVTMS samples fluorinated for 15, 30, 45, and 60 min.

The results of XEDS analysis of the content of the main elements over the thickness of the treated samples coupled with SEM microscale images of the film cross-sections showed that the distribution of the elements changed from the surface toward the depth of the films (Figure S2). The concentrations of elements on the very surface differed several times for XPS and SEM-XEDS (Figure 6 and Figure S2). For example, the oxygen content on the surface (at zero depth) for the sample fluorinated for 60 min was ~20 at. % (Figure 6, XPS data) against 5–6 at. % (Figure 8, XEDS data). This observation may be associated with the different spatial averaging of the element signal for both techniques. While the depth of the signal acquisition was ~1 nm in the case of XPS, the size of a spot at the cross-section of the film (perpendicular to the surface) was ~1 µm for XEDS [24,27]. This resulted in XEDS being less sensitive to changes in the concentration of elements and the elements’ concentration being lower for XEDS (presented in Figure 8 for carbon, silicon, and oxygen). In spite of the significant scatter of the concentrations for the XEDS technique (approximately ±5 at. % for carbon, ±3 at. % for silicon, and ±1 at. % for oxygen), one can notice a lower concentration for silicon (Figure 8b) and a higher concentration for oxygen (Figure 8c) at zero depth compared to the ones in the polymer bulk. It should also be mentioned that the concentrations of the elements at depths greater than 3–5 µm were comparable with those determined by XPS for the virgin PVTMS (Figure 6 and Figure 8).

**Figure 9:**
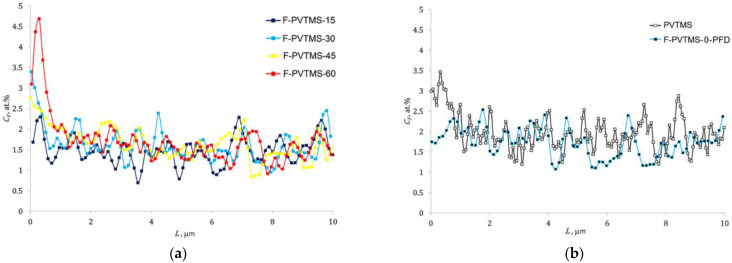
Fluorine content distribution over the thickness of the PVTMS film: (**a**)—depending on the fluorination time; (**b**)—for the virgin PVTMS sample and for the sample soaked in perfluorodecalin for 30 min (F-PVTMS-0-PFD).

Figure 9a shows a fragment (0–10 µm) of the distribution of the fluorine content (*C_F_*) along the thickness *L* for PVTMS samples fluorinated for 15, 30, 45, and 60 min. The equivalent fragment is shown in Figure 9b for the virgin film and for control sample soaked in PFD for 30 min.

The two following types of dependences can approximate experimental data for fluorinated samples:

(1) $$C_{F}=\exp\left(\frac{A\cdot t_{F}}{L}\right)\cdot C_{0}$$

and

(2) $$C_{F}=A\cdot t_{F}\cdot \exp\left(-\frac{L}{B}\right)+C_{0}$$

where *C_F_* is the atomic percentage of fluorine atoms; *C*_0_ is the constant coefficient that represents the background percentage of fluorine atoms; *L* is the distance from the treated surface of the sample, μm; *t_F_* is the fluorination time, min; *A* and *B* are constant coefficients.

Dependence (1) suggests a conditionally “infinite” content of fluorine (the absence of any other elements) on the treatment surface (*L* = 0) and the asymptotic tendency of this content to the background level (*C*_0_) in the polymer. The constant *A* in expression (1) has a physical meaning of the fluorine accumulation rate over the film thickness [μm/min] and carries information about the distance at which the value of the fluorine content will be e (the base of the natural logarithm) times greater than the background level after one minute of treatment.

Dependence (2) supposes the “finite” fluorine content on the treated surface of the film, which depends on the treatment time and asymptotically tends to the background level (*C*_0_) in the polymer. The constant *A* in expression (2) has a physical meaning of the fluorine accumulation rate [at. %/min] and is numerically equal to the increase (above the background level) of the fluorine content on the sample surface after one minute of its processing. The constant *B* [μm] is numerically equal to the distance from the treated surface at which the fluorine content (above the background level) decreases *e* times at a given treatment duration.

**Figure 10:**
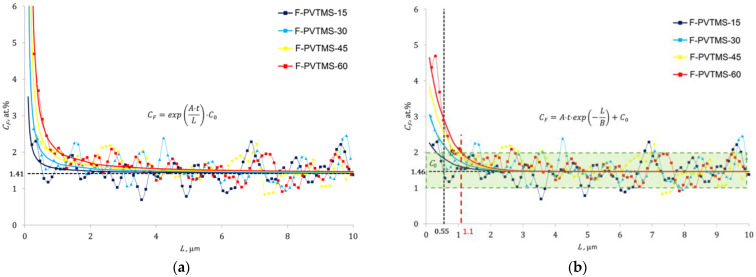
Approximation of the fluorine distribution over the film thickness for different fluorination durations: (**a**)—approximation by expression (1); (**b**)—approximation by expression (2).

The constant coefficients of Equations (1) and (2) were determined using experimental XEDS data and the least squares method (Table 1). Figure 10 presents the results of the approximation and their comparison with experimental points.

Prediction (with a technically sufficient degree of reliability) of the fluorine atom content at a given depth of a material after a given duration of its processing is possible using expressions (1) and (2), as well as the constant coefficients in Table 1.

The depth dependences (smooth curves based on Equation (1) or (2)) of the fluorine concentration for different fluorination times may facilitate an estimation of thickness of the fluorinated layer (i.e., the depth of fluorination). However, this estimation will not be precise due to the scatter of the concentration profile of fluorine, which corresponds, on average, to ± 0.5 at. % (Figure 10b). The transparent green rectangle in Figure 10b represents the scatter 1.46 ± 0.5 at. %, and its horizontal sides have intersections with the fitted lines (Equation (2)). The X-coordinates of these intersections correspond to the rough estimations of the depth of fluorine penetration for different durations of treatment. Therefore, the maximum depth of fluorination for the PVTMS sample fluorinated for 60 min is equal to ~1.1 µm (represented by the dashed red line in Figure 10b). This and lower depths of fluorination for the shorter durations are very close to the thicknesses of the fluorinated layers measured previously via the laser interferometry technique reported by Kharitonov et al. [15]. Therefore, one can conclude that fluorine penetrates into the PVTMS films to similar depths for the liquid- and gas-phase regimes of fluorination.

### 3.4. Contact Angle Measurements and Analysis of the Fluorinated PVTMS Samples

The results of the contact angle measurements for two different liquids (water and CH_2_I_2_) and the surface free energy components’ calculation are given in Table 2.

Data in Table 2 shows that fluorination of the PVTMS films for 15–45 min led to surface oxidation, i.e., the content of polar oxygen-containing groups in the surface layer became sufficient for surface hydrophilization. The dispersion component *γ^d^_SV_* of the surface free energy increased with the fluorination time, which indicates likely surface layer compaction.

Fluorination for 60 min led to an increase in the fluorine concentration in the surface layer and hydrophobization of the surface. It was suggested that oxygen-containing groups change their orientation toward the film bulk and contribute to the film surface layer compaction (leading to an increase in the dispersion component).

## 4. Conclusions

Polyvinyltrimethylsilane, a well-known material for gas separation, was treated by fluorine (F_2_ [10 vol.%] + N_2_) in the liquid perfluorodecalin for 15, 30, 45, and 60 min. The obtained unilateral surface fluorinated samples were investigated by various structural techniques (ATR-IR, SEM-XEDS, XPS, and the wetting measurement method). ATR-IR showed a gradual decrease in the intensity of the bands related to -CH_3_ (in SiMe_3_) and C-CH (in the backbone of PVTMS) and an increase in that of the Si-CH_2_ and C-F groups with an increase of the duration of treatment, which reflects an introduction of fluorine in the PVTMS chemical structure of the surface layer. Oxygen-containing groups were also detected and indicate that fluorination was accompanied by the oxidation of the surface of PVTMS samples. The XPS technique showed that the fluorine- and oxygen-containing groups significantly increased in the very surface layer (1–60 nm) with fluorination time in comparison with their bulk concentrations. The fluorination procedure also resulted in a simultaneous decrease in the carbon- and silicon-containing moieties, which may prove partial cleavage of C-Si bonds. According to XPS data, the average maximal content for five repeated units on the surface of PVTMS fluorinated for 45 and 60 min could be represented as 13 C, 1 Si, ~6 F, and ~5 O atoms. SEM-XEDS allowed us to make an estimation of the distribution of the elements (C, Si, O, F) forming PVTMS films along the depth of the film. The chemical content of the elements in the fluorinated layer as well as in the bulk of the PVTMS films was qualitatively consistent with that detected by the ATR-IR and XPS methods; however, the concentrations of the elements (against XPS) were lower due to the larger spatial averaging of the characteristic signal. The fluorine content CF evidently increased with the duration of fluorine treatment. Two exponential models with and without the intercept were suggested to describe the distribution of CF dependence on the fluorination time and the distance from the treated surface of the film. The model with the intercept helped to estimate the depth of fluorination for different treatment times: ≤1.1 µm for the films fluorinated for ≤60 min. Contact angle measurement demonstrated that during the fluorine treatment for up to 45 min, the hydrophobic surface of the virgin PVTMS film transformed to a semi-hydrophobic one. This was due to a predominant increase in the polar component of surface energy (accumulation of polar oxygen-containing groups). Further fluorination led to a sharp decrease in the polar component and the corresponding increase in hydrophobicity of the PVTMS film surface. The latter behavior can be explained by reorientation of the polar groups toward the bulk of the film. In general, the current investigation proved that liquid-phase fluorination differed due to lower attraction to the chemical structure of the fluorinated layers in comparison with the gas-phase regime that may allow more precise tuning of the surface-dependent properties.

## Figures and Tables

**Table 1:** Constant coefficients of Equations (1) and (2) for the studied films.

Equation	*A*	*B*, μm	*C*_0_, at. %
(1)	0.006 μm/min	-	1.41
(2)	0.064 at. %/min	0.551	1.46

**Table 2:** Characteristics of the surface of PVTMS films depending on the time of fluorination.

Fluorination Time, min	*θ*(water), deg	*θ*(CH_2_I_2_), deg	*γ^d^_SV_*, mJ/m^2^	*γ^p^_SV_*, mJ/m^2^	*γ_SV_*, mJ/m^2^
0	91	66	22	4	26
15	76	48	30	7	37
30	74	42	33	7	40
45	70	42	32	10	42
60	102	49	36	0	36

## Data Availability

Not applicable.

## Supplementary Materials

The following supporting information can be downloaded at https://www.mdpi.com/article/10.3390/ma16030913/s1, Figure S1: XPS spectra of C1s (a), Si2p (b) F1s (c), and O1s (d) lines for virgin PVTMS sample without and after Ar+ irradiation etching for 60, 180, 300, 600, and 900 s; Figure S2: Curves of the atomic content of fluorine along the cross-section surfaces of the samples treated for 15 (a), 30 (b), 45 (c), and 60 (d) min. The vertical green lines correspond to the zero-depth surface of the fluorinated PVTMS samples.
