# Supplementary material for: Surface and Structural Characterization of PVTMS Films Treated by Elemental Fluorine in Liquid Perfluorodecalin
Source: Materials (Basel). 2023 Jan 18;16(3):913. doi: 10.3390/ma16030913 (PMC9917527; doi:10.3390/ma16030913)
Supplement: Supplementary file 1 [file materials-16-00913-s001.zip › materials-2146835-supplementary.pdf]

Supplementary Materials

Surface and Structural Characterization of PVTMS Films Treated by Elemental Fluorine in Liquid Perfluorodecalin

Nikolay A. Belov [1,2,*], Aleksandr Y. Alentiev [1,2], Dmitrii S. Pashkevich [1,3], Fedor A. Voroshilov [1], Edgar S. Dvilis [1], Igor P. Asanov [4], Roman Y. Nikiforov [1,2], Sergey V. Chirkov [1,2], Daria A. Syrtsova [2], Julia V. Kostina [2] and Yulia G. Bogdanova [5]

[1] Tomsk Polytechnic University, 30, Lenin Avenue, 634050 Tomsk, Russia
[2] A.V. Topchiev Institute of Petrochemical Synthesis, Russian Academy of Sciences, 29, Leninskii Prospect, 119991 Moscow, Russia
[3] Institute of Applied Mathematics and Mechanics, Peter the Great St. Petersburg Polytechnic University, 29, Polytechnicheskaya St., 195251 Petersburg, Russia
[4] A.V. Nikolaev Institute of Inorganic Chemistry, Siberian Branch of Russian Academy of Sciences, 3, Academician Lavrentiev St., 630090 Novosibirsk, Russia
[5] Chemical Department, M.V. Lomonosov Moscow State University, GSP-1, Leninskie Gory, 119991 Moscow, Russia
* Correspondence: belov@ips.ac.ru; Tel.: +7-(926)-432-8323

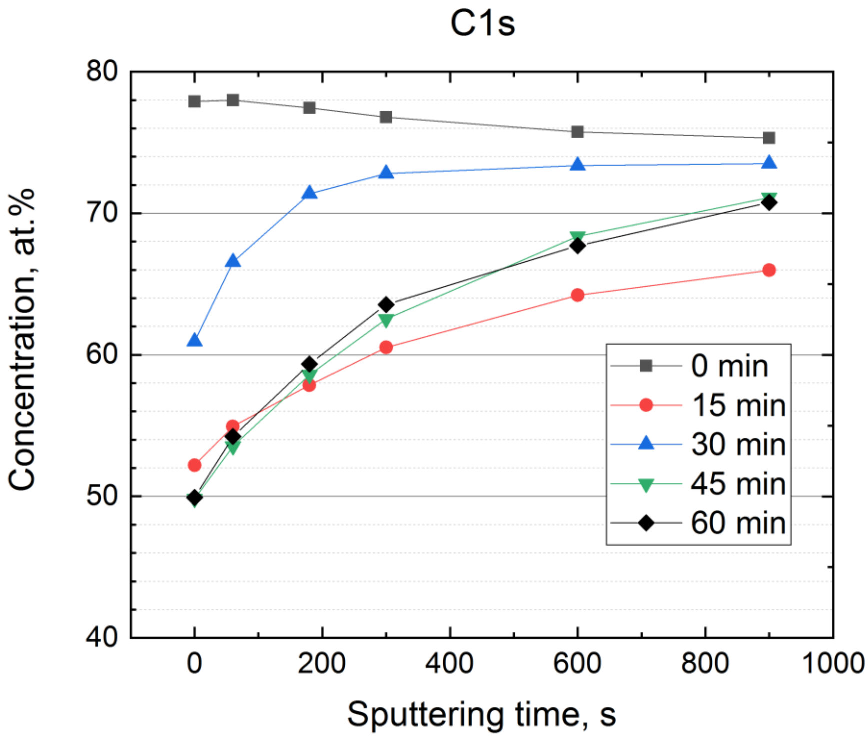
C1s
Concentration, at.%
Sputtering time, s
80
70
60
50
40
0
200
400
600
800
1000
0 min
15 min
30 min
45 min
60 min

(a)

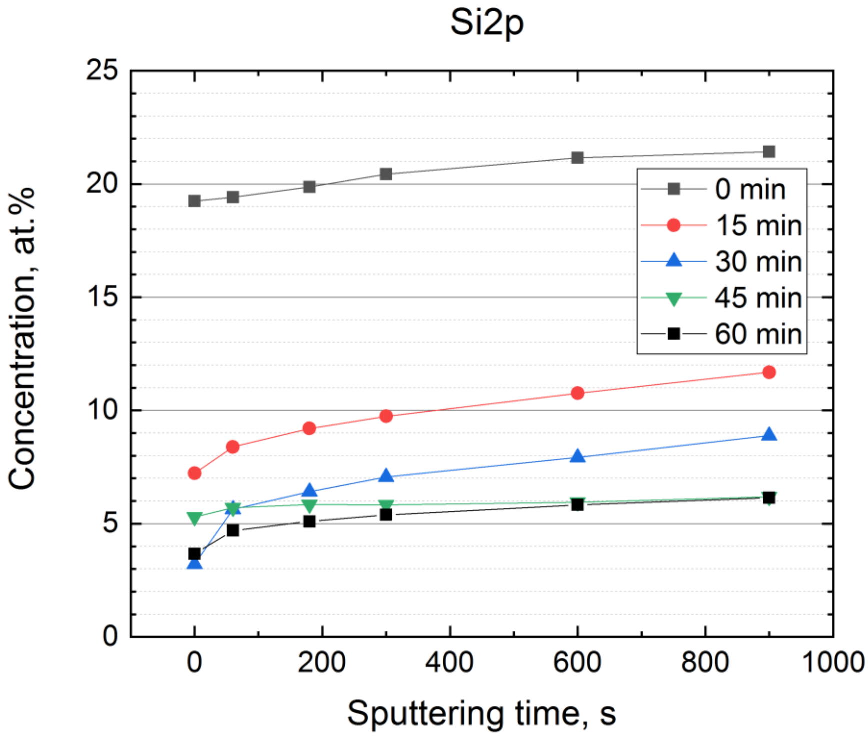
Si2p
Concentration, at.%
Sputtering time, s
25
20
15
10
5
0
0
200
400
600
800
1000
0 min
15 min
30 min
45 min
60 min

(b)

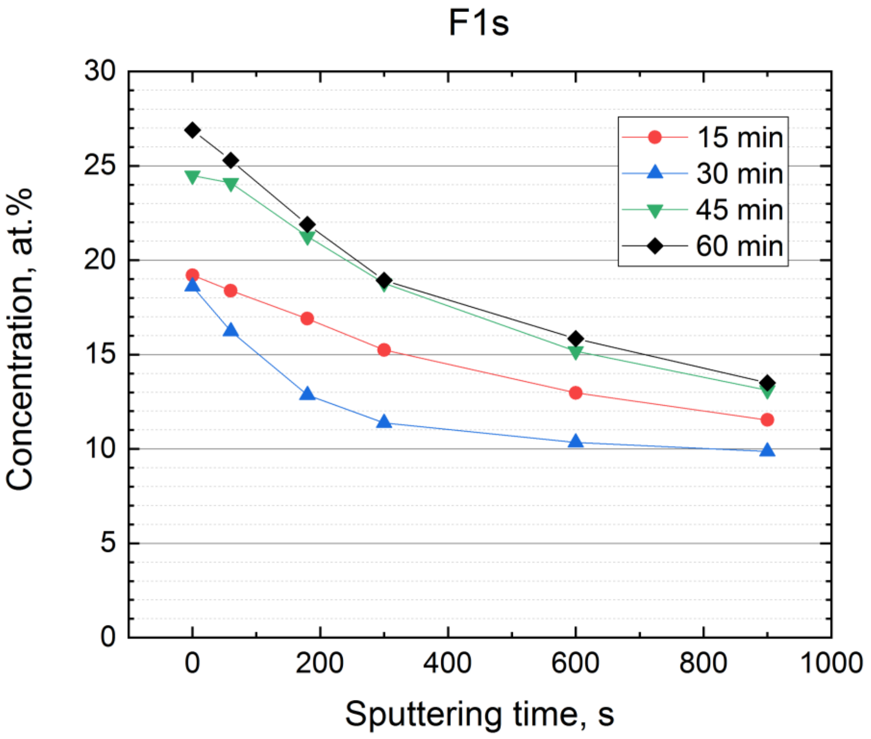

(c)

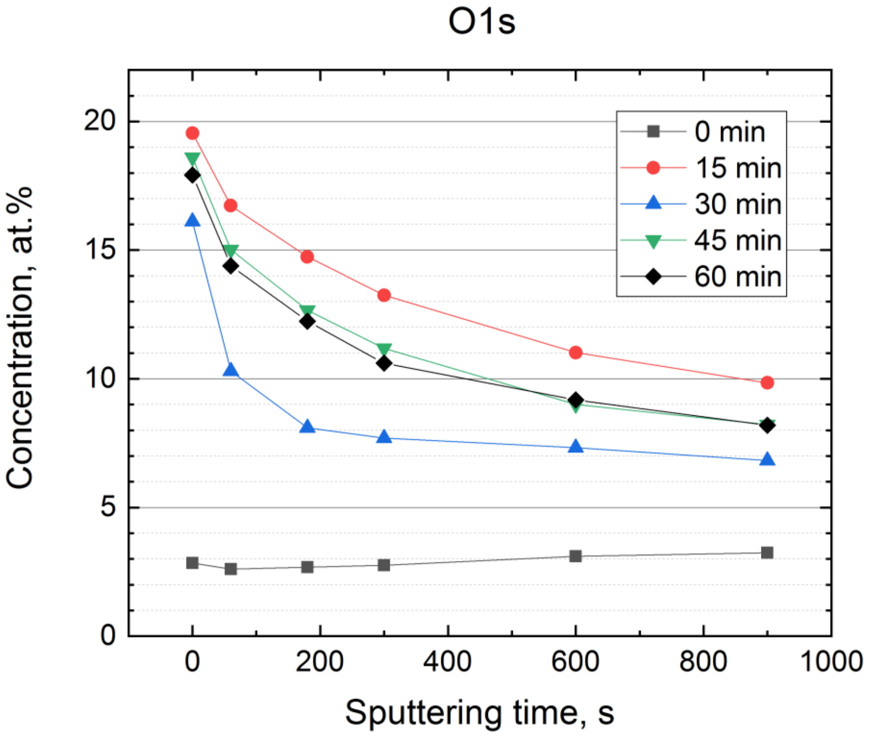

(d)

Figure S1. XPS spectra of C1s (a), Si2p (b) F1s (c) and O1s (d) lines for virgin PVTMS sample without and after Ar^+ irradiation ethcing for 60, 180, 300, 600 and 900 s

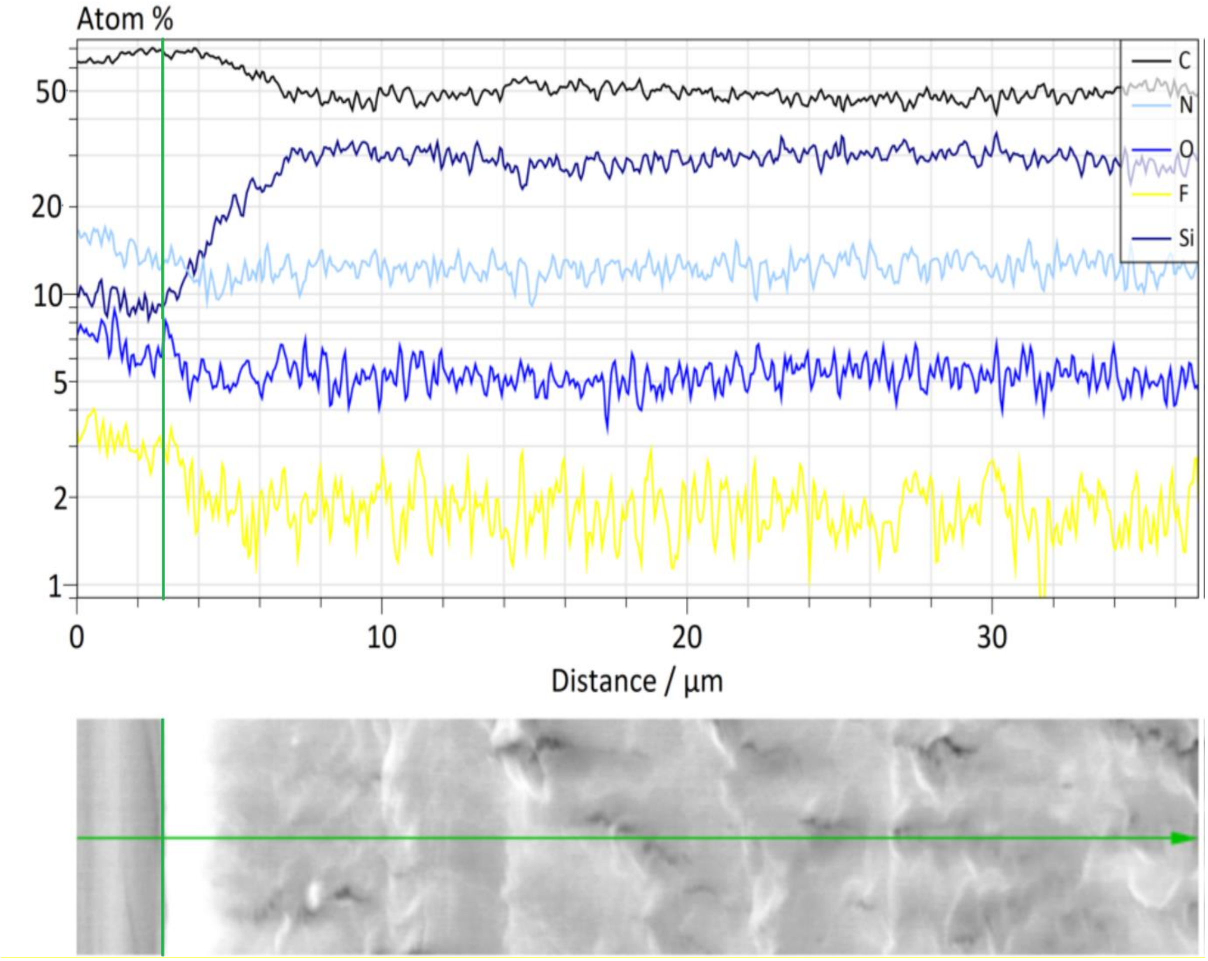

(a)

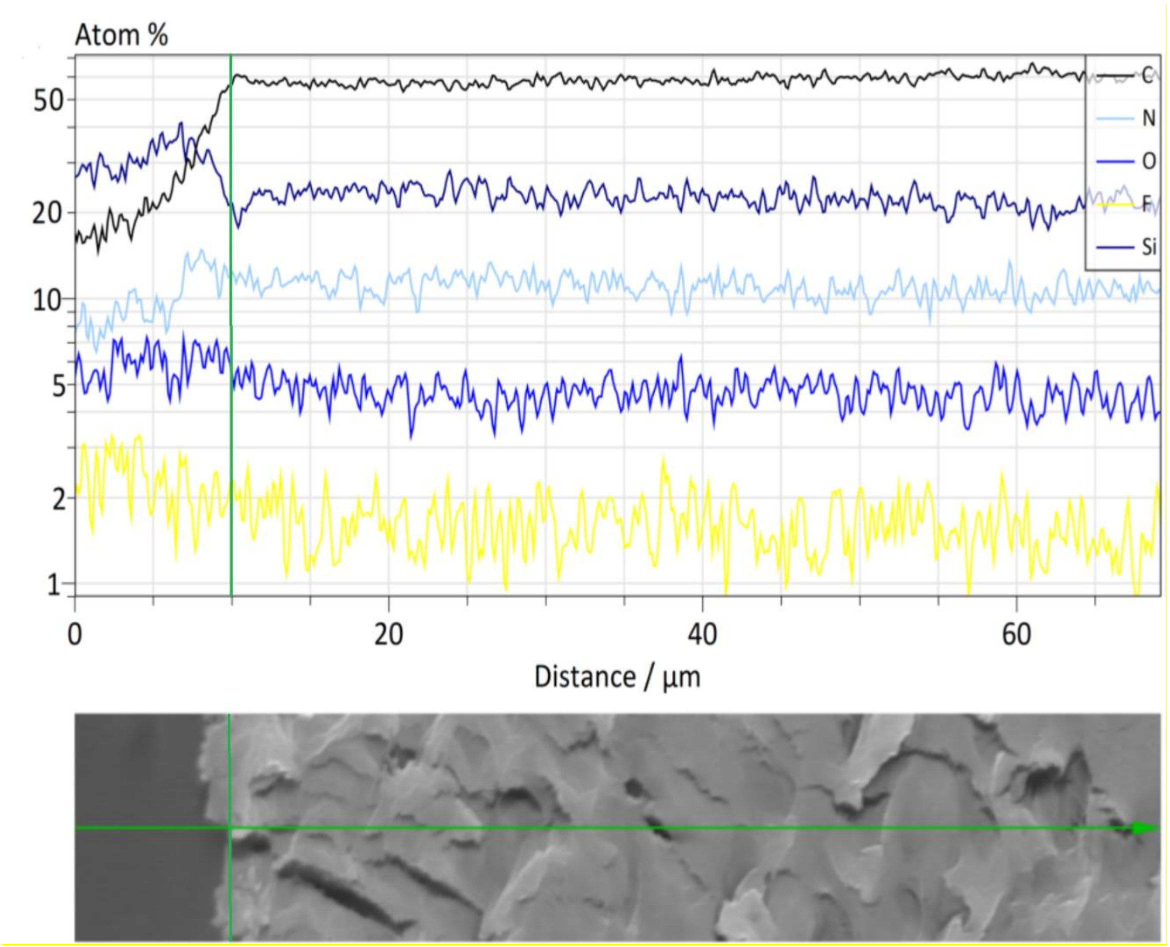

(b)

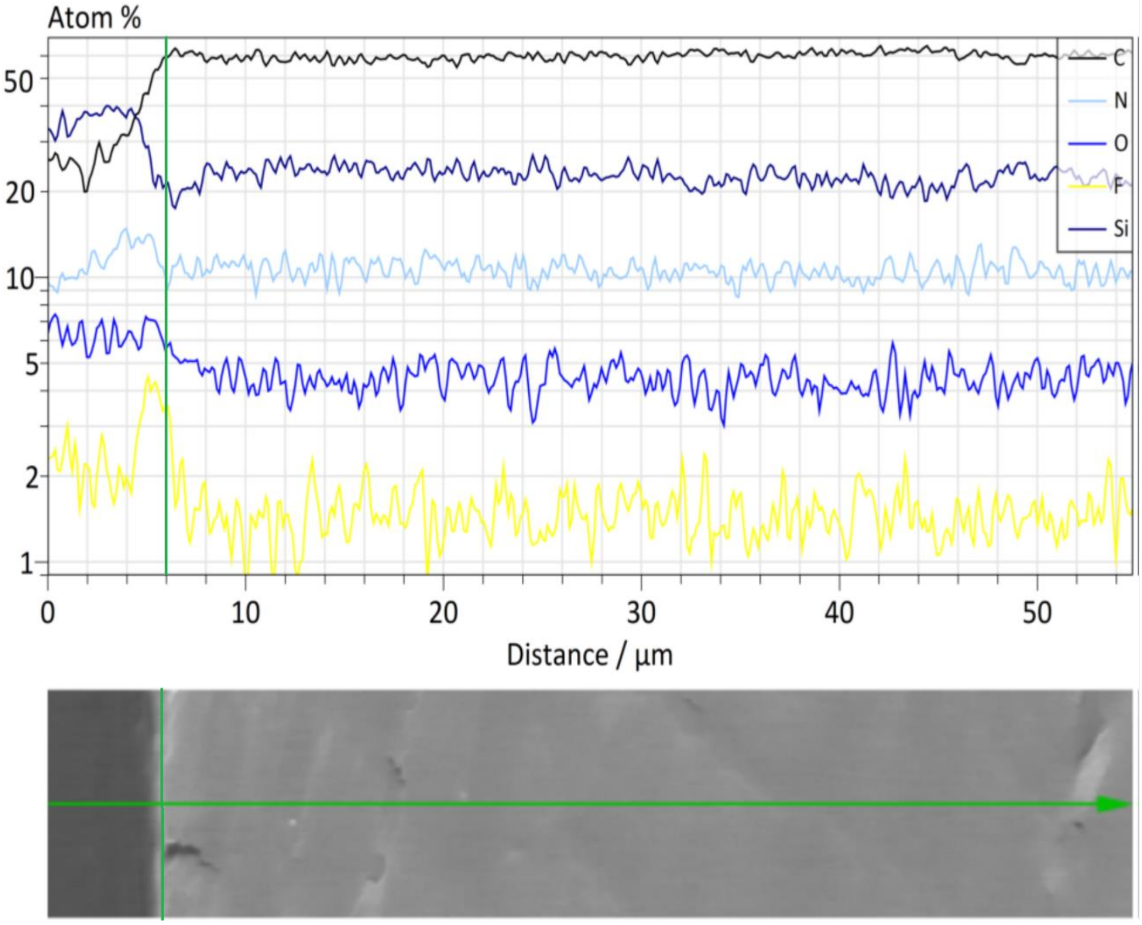
Atom %
50
20
10
5
2
1
0
10
20
30
40
50
Distance / µm
C
N
O
F
Si

(c)

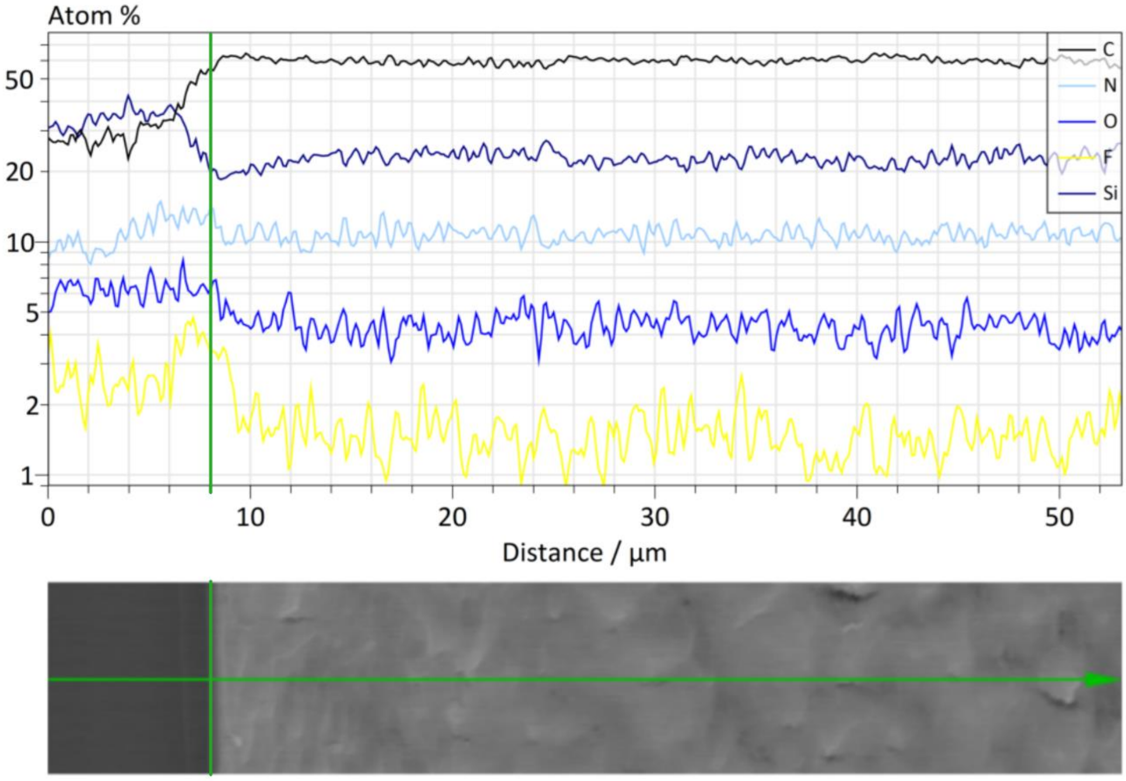
Atom %
50
20
10
5
2
1
0
10
20
30
40
50
Distance / µm
C
N
O
F
Si

(d)

Figure S2. Curves of the atomic content of fluorine along the cross-section surfaces of the samples treated for 15 (a), 30 (b), 45 (c), and 60 (d) min. The vertical green lines correspond to the zero-depth surface of the fluorinated PVTMS samples
